# Effects of Insecticides and Microbiological Contaminants on *Apis mellifera* Health

**DOI:** 10.3390/molecules26165080

**Published:** 2021-08-22

**Authors:** Aleksandra Leska, Adriana Nowak, Ireneusz Nowak, Anna Górczyńska

**Affiliations:** 1 Department of Environmental Biotechnology, Lodz University of Technology, Wolczanska 171/173, 90-924 Lodz, Poland; 2 Faculty of Law and Administration, University of Lodz, Kopcinskiego 8/12, 90-232 Lodz, Poland; inowak@wpia.uni.lodz.pl (I.N.); agorczynska@wpia.uni.lodz.pl (A.G.)

**Keywords:** *Apis mellifera*, colony collapse disorder, bee pathogens, pesticides

## Abstract

Over the past two decades, there has been an alarming decline in the number of honey bee colonies. This phenomenon is called Colony Collapse Disorder (CCD). Bee products play a significant role in human life and have a huge impact on agriculture, therefore bees are an economically important species. Honey has found its healing application in various sectors of human life, as well as other bee products such as royal jelly, propolis, and bee pollen. There are many putative factors of CCD, such as air pollution, GMO, viruses, or predators (such as wasps and hornets). It is, however, believed that pesticides and microorganisms play a huge role in the mass extinction of bee colonies. Insecticides are chemicals that are dangerous to both humans and the environment. They can cause enormous damage to bees’ nervous system and permanently weaken their immune system, making them vulnerable to other factors. Some of the insecticides that negatively affect bees are, for example, neonicotinoids, coumaphos, and chlorpyrifos. Microorganisms can cause various diseases in bees, weakening the health of the colony and often resulting in its extinction. Infection with microorganisms may result in the need to dispose of the entire hive to prevent the spread of pathogens to other hives. Many aspects of the impact of pesticides and microorganisms on bees are still unclear. The need to deepen knowledge in this matter is crucial, bearing in mind how important these animals are for human life.

## 1. Introduction

A semi-free-ranging species of *Apis mellifera* is valued all over the world for numerous honey properties and ecological importance in reproduction of plants. It also plays a significant role in the pollination of many economically important crops [1]. 

Honey bees are complex eusocial insects and their association with people has lasted for at least 7000 years [2]. The European honey bee has spread throughout the world thanks to the activities of beekeepers, however, the native range of *A. mellifera* is even more diverse and covers Europe, the Middle East, and Africa. Phylogenetic analysis based on mitochondrial markers and nuclear DNA support the classification of *A. mellifera* as cavity-nesting bees. Based on this fact, it can be concluded that the European bee originates from Asia, similar to other species belonging to this group [3]. 

Bees live in tight societies where each individual is responsible for the development and survival of the colony. The organization of a bee colony shows many analogies to a multicellular organism (often referred to as the “superorganism”). An example of such an analogy is that both a bee colony and an organism are made up of individual units. In the case of bee colonies, such units are bees, and in organisms, cells. Both bees and cells depend on bee colonies and organisms, respectively, and are unable to develop properly outside of them.

Bees are closely connected to human well-being by affecting the ecosystem, crop production, food safety, and the reproduction of wild plants, providing livelihood security and reduce poverty among urban communities and rural areas through beekeeping based on local knowledge. Due to the importance of the topic and the problem, this article addresses issues related to the harmful effects of various pesticides on bees’ health. The article also discusses the problem of microorganisms that negatively affect bee colonies’ health and contribute to the Colony Collapse Disorder (CCD). This phenomenon is particularly significant when taking into consideration the importance of bees in nature and the positive impact of bee products on human health. Furthermore, the European Union’s legal protection for bees against pesticides is discussed. Understanding the great threat to bees from microorganisms and pesticides, it appears that finding a solution to the threat of the extinction of bees is crucial.

The authors of this article, in their previous publications, raised issues related to combating the infectious disease of honey bees, which is American foulbrood (*Paenibacillus larvae*), in the light of Polish legal regulations. In their previous publications, the authors also presented the characteristics of lactic acid bacteria (LAB) as an important part of the gut community and their special beneficial activities for honey bee health. The authors also discussed the idea of probiotics for honey bees as a promising tool to improve their health [4,5].

## 2. The Significance of Bees to Humans as an Economically Important Species

Apiculture requires minimal investment, often occurs without land ownership, and generates various products for later sale [6]. Bees are therefore an economically important species. The long-term survival of farming and agriculture around the world depends on insect pollination. This is connected with huge amounts of money reaching hundreds of billions of dollars a year [7]. It is increasingly known that pollination also affects the nutritional value of food. The lack of macronutrients and numerous essential micronutrients cause specific conditions of nutrient deficiency, as well as greatly increase mortality from other diseases or weaken the immune system and the development of stunts [8].

Bees have a strong influence on ecological aspects, the preservation and stability of the ecosystem, biodiversity, and the genetic variation in the plant community. They play a significant role in most terrestrial ecosystems where the green vegetation lasts for at least 3 months a year. In tropical forests, savannah, and mangroves, many plants and animals would not survive without the existence of bees. This is as bees are the main pollinators, and have a significant influence on the production of seeds and fruits. In cultivated areas, bees are needed to pollinate many crops and to preserve biodiversity on non-cultivated areas. Other animals are associated with bees by eating honey, pollen, or wax, being parasitic for the bees, or living in the bees’ nest [9].

International transport of the honey bee by humans has led to its current cosmopolitan spread, which spans all continents except some oceanic islands and Antarctica. Having such an advanced level of knowledge about this species, it seems unforeseen that the role of bee as a pollinator in natural habitats is still poorly understood [10]. It was also found that the number of managed honey bees, *A.* *mellifera*, and wild bees is equally important in describing the results of open pollination [11].

Precise predictions of pollination performance require a thorough understanding of the interactions between plants and flower visitors [12]. It is known that at least 75% of the 115 leading crop species benefit from animal pollination [13]. A decrease in bee pollination service could potentially reduce yields by approximately 40% [6].

However, populations of wild and managed pollinators are declining. Research conducted in western Europe also showed unprecedented dismutation in both biomass and biodiversity of wild pollinators among many taxonomic groups. For this reason, many European countries have decided to develop pollinator maintenance strategies in the context of the *European Initiative* and *Willing on Pollinators*. The approach to pollinator maintenance differs between European countries, and the focus of the conservation strategy varies from country to country, e.g., in Belgium the initiative is dedicated exclusively to honey bees, and in France to all pollinators [14]. 

## 3. Products of Bee Origin—Effect on Human Health, Its Various Applications, and a Source of Chemical Contaminants

Honey is a sweet food product produced by honey bees by processing the flower nectar of plants, as well as some secretions found on tree leaves. Honey is used to treat throat infections, hiccups, dizziness, bronchial asthma, worm infection, tuberculosis, and as a nutritious supplement. It is also used for wounds in traditional medicine [15]. Raw honey can have a positive impact on oral well-being and dental health due to its antibacterial activities that work against both Gram positive and Gram negative bacteria [16]. Honey ingredients have anti-inflammatory [17] and antiproliferative properties [18]. Due to this, honey has the potential to treat many diseases and ailments such as diabetes [19]. Additionally, the two main biologically active ingredients of honey are flavonoids and polyphenols, which are known for their antioxidant abilities [20]. Hydrogen peroxide is produced enzymatically and is an important factor in honey’s antibacterial activity. This compound is produced due to the glucose oxidase, which occurs naturally in honey and is active only after dilution. Glucose oxidase is inactive at low pH of honey, which is caused by the presence of organic acids (e.g., glucuronic acid) [21]. Another property of honey is its antifungal activity associated with the presence of methylglyoxal, glucose oxidase, and high sugar content [22].

Bee pollen is a bee product that has a positive effect on human health [23]. It is a mixture of honey bee secretions, nectar and plant pollen pellets. Bees use bee pollen in order to make bee bread. It contains such nutritional components as proteins, minerals, vitamins, polyphenols, and carbohydrates [24]. Bee pollen contains phytosterols phospholipids and unsaturated fatty acids due to which it exhibits hypoglycemic activity [25]. Flavonoids, polyphenols, and phenolic acids contained in bee pollen play an important role in preventing toxication and protecting the liver from toxins. The high content of omega fatty acids and essential amino acids strengthens the immune system, helps fight bacteria, and stimulates tissue repair [26]. Bee pollen also has anticancer activity due to the high content of biochemical components such as carotenoids and phenolics [27]. Bee pollen helps with malnutrition, which can often contribute to the deterioration of protein metabolism, digestive tract alterations, and immunological abnormalities [28]. It also has a positive effect on the skin by reducing water loss and influencing the lipid barrier. Bee pollen is also used to treat skin burns due to the high content of biologically active substances [29].

Propolis is a natural mixture produced by honey bees from substances collected from plants. Due to its mechanical properties and waxy nature, bees use propolis to build hives and provide thermal isolation by sealing cracks. In nature, it is hard and lipophilic, however, when heated, it becomes soft, sticky, and gummy [30]. Propolis has a positive effect on the immune system and exhibits many beneficial properties due to the content of bioactive constituents. The content of compounds varies depending on where the propolis was produced. The molecules contained in propolis include, but are not limited to, esters, flavonoids, amino acids, aldehydes, phenolic acids, vitamins, fatty acids, and minerals [31]. Flavonoid content inhibits formation and attachment of biofilms and metabolism energy of bacteria [32]. These compounds are associated with antibacterial activity of propolis and thus have found their use in the treatment of oral diseases [33]. Flavonoids are also strong antioxidants, able to protect the cell membrane against lipid peroxidation and have a positive effect on oxidative stress [34]. 

Royal jelly is a mandibular and hypopharyngeal secretion secreted by worker bees. Bees use royal jelly to feed larvae and to help nature the brood. Royal jelly is also food for the queen, and it makes her live longer than the rest of the colony [35]. The composition of royal jelly includes vitamins, proteins, water, carbohydrates, mineral salts, and lipids [36]. It has the potential to treat human diseases due to its antioxidant, antiaging, antitumor, and anti-inflammatory activity [37,38,39].

Due to human activity, trace amounts of toxic molecules can be detected in bee products. Honey and royal jelly can often be contaminated with antibiotics that are used in agriculture to fight harmful pathogens (e.g., chloramphenicol chlortetracycline and doxycycline). Some antibiotics produce hypersensitivity and can also directly trigger toxic reactions and weaken the immune system of the bees and consumers [40]. Pesticides are chemical impurities that can be detected in bee products (especially propolis and pollen) and a lot of them are hazardous. The most common insecticides contaminating bee products include neonicotinoids, organochlorines (e.g., coumaphos and chlorpyrifos), organophosphates, and carbamates [41,42,43,44]. Honey products and bee colonies can be also contaminated by heavy metals contained in air and soil. Examples of such impurities are cadmium, zinc, and copper. Heavy metals, absorbed above the levels of pollution standards, can also threaten bee and human health [45,46]. Other chemical contaminants, the residues of which may be present in bee products, are polychlorinated biphenyls and polycyclic aromatic hydrocarbons (congomers and mixtures) [47,48,49,50,51,52,53,54]. Knowing this, it can be concluded that bee products, through their chemical contamination, can negatively affect bee and human health, and this is an urgent problem of modern beekeeping. Due to the above, honey can be used as a bioindicator of environmental pollution. 

## 4. Colony Collapse Disorder and Factors Presumably Causing It

During the winter of 2006/2007 a mysterious decrease in the number of bees in colonies was observed in Europe and the United States [55,56]. It was determined by the sudden decline in adult worker bees in the colonies, for no specific and obvious reason. This phenomenon was called “Colony Collapse Disorder” [56]. In 2006–2016, the winter loss varied in the range of 21.9–35.8% and the average decrease in the number of bee colonies was estimated at 28.7% [57]. Statistics by the United States Department of Agriculture (USDA) report that honey bee colonies lost with CCD symptoms on operations with five or more colonies was 105,240 colonies from January through March 2020, which is a 76% increase from winter 2019 [58]. In recent years, rates of loss linked to CCD have declined despite continued high rates of winter losses. It should not be forgotten, however, that many other factors (e.g., starvation, mismanagement, queen problems, and parasites) can influence the mortality of bee colonies and the overall mortality of bees is not entirely caused by CCD [59,60]. Furthermore, over the past fifty years, the number of managed bee hives has increased worldwide, even though there have been declines in some European countries and North America [61].

**Figure 1:**
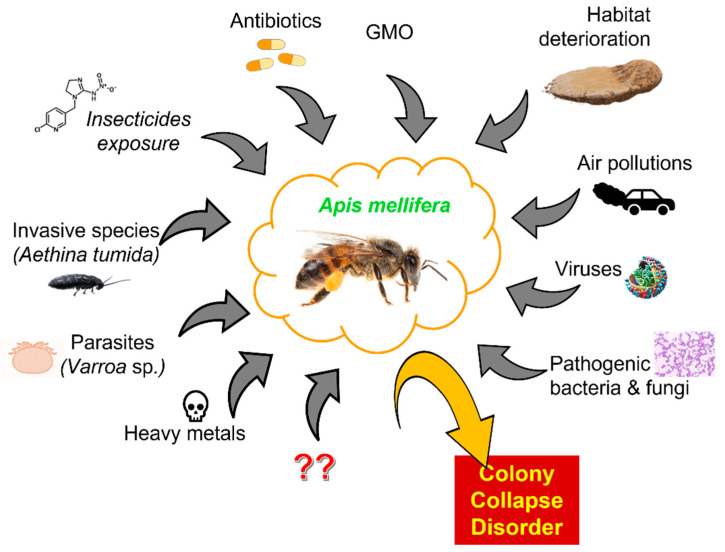
Factors contributing to CCD of *A. mellifera*.

Given the importance of pollinators in relation to global food, the decline in bee numbers is at least worrying [62]. After the collapse of a colony, immature bees and food remains are often found in the hives. This means that the death of worker bees was probably sudden and early [63]. Pollinators affected by CCD show many changes in rectum symptomology, which may indicate that these individuals have a problem with water regulation [64]. This phenomenon can be caused by various reasons. One of them is habitat deterioration. Human activities led to degradation, fragmentation, and destruction of natural habitats. Changes in the landscape structure affected bees and pollinated plants. Degradation of the natural environment of pollinators caused a reduction in the amount of bee food and changes in the bee population [65]. Another factor contributing to the decline in the number of bee colonies is GMO, which may have the potential for sub-lethal effects on bees, thus disrupting their feeding behavior [66]. Viruses are a known health threat to bees. In some cases, they can cause lethal diseases and lead to the death of an entire colony. Viruses can infect bees at various stages of development and drastically shorten their lives [67]. Invasive species are another factor. An example of such a species is, for instance, a small beetle inhabiting hives (*Aethina tumida* species), which colonized Australia and North America and is a threat to Europe, as its larvae destroy stored pollen and honey [68]. Another invasive species that pose a huge threat to bees are wasps that steal their larvae, honey, and adult individuals to provide protein and sugar for their brood and themselves [69]. Another factor triggering CCD is air pollution, which hinders the relationship between flowers and pollinating insects. Pollution affects the chemical components that plants use to lure insects which, as a result, makes it difficult for bees to locate food sources [70]. Chemical compounds (e.g., pesticides, antibiotics, heavy metals), microorganisms, and parasitic mites are very crucial driving forces of pollinator population instabilities, which will be discussed in more detail later in this review. They significantly affect bee health and contribute to CCD. Their negative impact on bees causes a drastic decrease in the number of pollinators in the colony [63,71]. It is important to emphasize that, in most cases the factors mentioned above do not work in isolation. Usually, the interactions between them lead to damage and differ in different parts of the world [72]. Some factors influencing CCD are presented in Figure 1. They were reviewed in detail by Hristov et al. [73].

## 5. Pesticides in a Bee Environment and Their Consequences

### 5.1. Impact of Pesticides on Living Organisms and the Environment

Pesticides are biological agents or synthesized substances used for killing or restricting the development of organisms [74]. Pesticides include fungicides, herbicides, insecticides, and rodenticides. In animals and humans’ bodies, pesticides are metabolized, stored, excreted, and bioaccumulated in body fat. Pesticides can enter the body through various routes, e.g., inhalation, absorption through damaged skin, or ingestion [75]. Exposure to pesticides can be associated with numerous negative health effects such as dermatological, gastrointestinal, carcinogenic, respiratory, reproductive, and neurological effects. Long-term exposure to pesticides can cause chronic effects on health [76]. 

For a long time, pesticides have been suspected as one of the main reasons for the decline in bee colonies [77,78]. High-quality seed technologies have led to even greater development of pesticides to protect plants seasonally, which leads to contamination of nectar and pollen. By contact with contaminated plants, bees can be exposed to substances that are harmful to them [79]. The damage pesticides can do to bees includes, but is not limited to, delayed development, impairment of immunity system, and shortening the life span of adults [80]. The honey bee genome contains far less annotated genes than the genomes of other insects. The genome of *A. mellifera* contains only about 11,000 genes coding for proteins, when the malaria mosquito *Anopheles gambia* has about 14,000 of them. Honey bees have half as much glutathione-s-transferases (GSTs), carboxyl/cholinesterases (CCEs) and cytochrome P450 monooxygenases (P450s). These enzymes are associated with resistance to insecticides in other species and the shortfall of them may cause pesticides sensitivity in bees. This also affects their susceptibility to pesticide activity, and may weaken their ability to fight virus and bacteria in the future [81]. Immature bees are less likely to be exposed to pesticides as they do not leave the hive. Older bee individuals may come into contact with contaminated pollen or nectar and transfer the impurities to the hive, which is then associated with infecting other individuals. Referring to this, pesticides threaten bees regardless of their age [82]. Queens and other bees are exposed to pesticides by contact with contaminated food and wax. Exposure to pesticides through contaminated wax can negatively affect reproduction of bees, e.g., reduce the number of eggs laid, reduce ovarian weight in queens, or increase queen cell rejection. Pesticide residues can accumulate in the wax for years and may migrate through the brood comb wax, contaminating an even larger area of the hive. Developmental exposure of honey bees to brood combs contaminated with pesticides may appear subtle, but can result in sub-lethal effects that have severe consequences [83]. 

Honey bees dedicate a large amount of their resources to the production of drones, which help in mating with virgin queens from neighboring colonies during the reproductive season. There are significant differences in the production of viable sperm cells that can fertilize an ovule between sexually mature drones that are exposed to various environmental conditions during their development or as adults. Pesticide contamination of beeswax adversely affects the reproductive quality of drones, which can also affect the queens they mate with, and ultimately weaken the health of the colony [84]. Fungicides and insecticides can also alter insect mobility, navigation, orientation, overall development, and immune function in bees [85]. 

Exposure to pesticides (e.g., acaricides) may also result in susceptibility to some parasites that threaten the health of the colony [86]. This may be due to pesticide-induced changes in the pathways of the immune system. Exposure to parasites can cause not only the death of certain individuals but also lead to the extinction of the entire colony [87]. The use of pesticides can also affect the bee’s environment. The intensive use of conventional pesticides can reduce the harvest network for bee colonies. These effects are exacerbated by the loss of natural habitat, which can make farms more dependent on pesticide inputs as natural pest control is lost, ultimately reducing pollinator pools [88]. The exposure of bees to pesticides during pollination of flowering crops is associated with both the density of crops in the landscape and the collection of pollen from focal crop. A significant amount of pesticides that create a danger to bee health most likely come from the remains found in pollen from non-focal crops, e.g., wild flowers [89]. This review article focuses on insecticides and their effects on the viability and health of bees. Particular attention was paid to neonicotinoids, fipronil, coumaphos, and chlorpyrifos due to their effect on honey bee health. Additionally, spinosad was discussed as an example of a biopesticide used in agriculture.

### 5.2. Insecticides Present in the Honey Bee Environment and Their Effects

#### 5.2.1. Neonicotinoids

Neonicotinoids are a globally used acetylcholine-interfering neurotoxic class of insecticides [90]. Acetylcholine is an excitatory neurotransmitter and endogenous agonist of the cholinergic nervous system. Neonicotinoids act as agonists on the nicotine receptors of acetylcholine (nAChR). In insects, the AchR is predominantly distributed in the neuropil regions of the central nervous system, which is responsible for rapid neurotransmission [91]. Neonicotinoids include compounds such as imidacloprid, thiamethoxam, and clothianidin [92]. Neonicotinoids are used, among others, in urban landscaping, veterinary medicine and to protect crops in agriculture. Neonicotinoids target parasitic sucking insects, soil insects, and crop feeding parasites. In veterinary medicine, they are used to kill fleas on pets [93]. The application of neonicotinoids is varied, but the most common method is to use them as a soil or seed treatment [94]. Neonicotinoids are water-soluble, moderately small molecules. After absorption by the plant, neonicotinoids and their metabolites reach various plant tissues [95]. Neonicotinoids can be found in nectar and pollen collected by pollinators. Neonicotinoids increase the mortality rate of a bee colony and contribute to the reduction in its social immunity [96]. Bees show symptoms of pesticide poisoning including convulsions, uncoordinated movements, and tremors. This negatively affects the condition of bees, weakening their health and the ability to learn, forage, and remember flower locations [97]. Neonicotinoids have synergistic and additive effects on honey bees, together with stressors such as nosemosis. In addition, some genes responsible for bees’ detoxification, immunity, behavior, and nutrition are up regulated by pollen or pesticide stress. The proper nutrition of bees is of great importance. The combination of exposure to pesticides, along with poor nutrition, can lead to unfavorable effects [98]. 

Exposing queens to neonicotinoids may result in a reduction in genetic diversity in bee colonies. The influence of neonicotinoids may affect mating of the queen before the formation of a new colony, which has a very negative effect on the health of the entire colony [99]. Bees are more likely to be exposed to neonicotinoids during planting season due to the high concentrations of these pesticides caused by the spread of these chemicals during and after planting [100]. 

#### 5.2.2. Coumaphos

Coumaphos is an organophosphate-based acaricide and a stable lipophilic compound. It is mainly used to control livestock pests and insects including lice, mosquitoes, fleas, ticks, and flies [101]. Coumaphos is an acetylcholinesterase inhibitor that targets cholinergic signaling and covers most excitatory neurotransmission in the nervous system of parasites. It occurs in the form of a crystal with a slightly brownish color and a slight sulfuric odor [102]. Coumaphos is also used to control varroosis in bee colonies in the form of liquid and strips. Coumaphos may negatively affect health of the bee colony. The low toxicity of coumaphos at least partially depends on rapid detoxification mediated by the P450s. The synergism of coumaphos and, e.g., fluvalinate, causes antagonistic interactions as both compounds are metabolized by P450s. This increases the toxicity of each of these compounds to potentially harmful levels for bees [103]. Drones exposed to coumaphos have reduced body weight and higher mortality. Coumaphos also affects the expression of genes for the detoxification pathways and can lead to a decrease in the level of bee gene products associated with hormonal and cellular immunity. It affects immune responses, physiological and detoxification functions in bees, making them more susceptible to other pesticides and pathogens [104]. Continuous exposure to this pesticide may result in reduced foraging activity and affect the size of hypopharyngeal glands. Coumaphos spreads across the colony mostly by physical contact between the nest partners [105]. Trace concentrations of coumaphos may occur in beeswax, honey, and bee brood. Concentrations of this pesticide can be also determined in beeswax in beehives where coumaphos has not been used, which is probably due to the spread of this pesticide by bees. Coumaphos can accumulate in beeswax for up to 5 years and the larvae exposed to this pesticide are characterized by delayed larval development [106].

#### 5.2.3. Chlorpyrifos

Chlorpyrifos is a triphosphorous organophosphate insecticide of wide commercial use. It targets such pests as cockroaches, ticks, and fleas, and has found its application in horticulture, viticulture, forestry, and agriculture. Chlorpyrifos is found in a variety of formations such as wettable and granular powders, micro-encapsulated suspensions, gel-based products, and emulsifiable concentrates [107]. Chlorpyrifos, similar to other organophosphate insecticides upon bioactivation, inhibits acetylcholine in the brain and the peripheral nervous system, causing neurotoxic effects in pests and non-targeted organisms. Inhibition of acetylcholinesterase results in a reduction in acetylcholine degradation and, consequently, overstimulation of associated synapses [108].

Chlorpyrifos also has toxic effects on insects beneficial for the environment, including honey bees, by inhibiting acetylcholine in their nervous system. Under the influence of exposure to chlorpyrifos, adult bees suffer from memory and learning disorders. Chlorpyriphos induces both slowed acquisition and odor generalization in bees of foraging age. Honey bees have difficulty finding their way to the hive and the flowers, which is the reason they are less efficient in collecting food for the colony. Chlorpyrifos increases larval mortality of the colony and can be detected in bee products such as nectar, propolis, wax, and pollen [109]. In addition, sublethal levels of chlorpyrifos interfere with development of the queen, which negatively affects reproduction of bees in the colony [110].

#### 5.2.4. Spinosad

Spinosad is an insecticide obtained from actinomycete bacteria *Saccharopolyspora spinosa.* The fermentation of bacteria produces metabolites that are part of this pesticide. The major components of spinosad are spinosyn A and spinosyn D [111]. It is mainly sold as water-dispersible granules or as a water-based suspension concentrate. Spinosad activates the nicotinic acetylcholine receptor in insects, but at a different site from neonicotinoids. Spinosad also affects the γ-aminobutylic acid receptor, but its role in overall linkage activity is ambiguous. Spinosad has a broad spectrum of activity on key pests, favoring the environmental profile and efficiency. It is used to kill insects such as leaf miners, thrips, and caterpillars, who destroy, for example, cabbage, spinach, and tomato crops [112]. Spinosad is environmentally friendly as it is degraded by the microbial action of the soil. It poses a minor threat to bee health and beehive activity. In laboratory conditions, the use of spinosad does not affect colony mortality. However, freshly sprayed, it may be intrinsically toxic to bees, and sprayed pollen and nectar may be harmful to brood development. To avoid this, spinosad should be allowed to dry on plant foil for about 3 h. Dry residues are not toxic and do not affect the honey bee’s viability [113]. 

#### 5.2.5. Fipronil

Fipronil is a phenylpyrazole insecticide often used to control insects including fleas, termites, cockroaches, and mosquitoes. Thanks to its wide application, it is used both on animals and plants. Fipronil is a chiral molecule due to the presence of the asymmetric sulfur atom and each enantiomer exhibits a different toxicity. In most cases, S-fipronil is less active against target organisms than R-fipronil. Fipronil interfere with the function of the ɣ-aminobutylic acid receptor chloride channels. This insecticide disrupts the flow of chlorine ions causing ɣ-aminobutylic acid to build up in synaptic junctions. After application of appropriate doses, fipronil, due to its mode of action, leads to the hyper-expression of the insect nervous system, paralysis, and death [114]. As with neonicotinoids, chemical residues of fipronil can be found in pollen and nectar due to its systemic properties. The fipronil molecules can be taken up by the roots from the soil water and spread throughout the plant, reaching its various tissues [115].

Fipronil may also affect the health of non-target insects, including honey bees. Fipronil is an inhibitor in the mitochondrial bioenergetics of bees, which causes ATP depletion and activation of glycolysis [116]. Fipronil can also cause morphological alterations in larvae midgut, leading to the vacualisation of the cytoplasm, and promote defects in the respiratory process, thereby disrupting the neural activity of bees. This neurotoxic phenylpyrazole insecticide, when ingested by bees, often causes agitation, convulsions, and paralysis. It also often interferes with motor activity, leading to a reduction in yields in crops pollinated by bees [117]. Fipronil also affects the number of hatched eggs, while reducing the number of new worker bees [118]. This insecticide is known to induce lethal and sub-lethal effects in bee colonies, thereby weakening their immune system and increasing bee mortality [117].

#### 5.2.6. Possible Effects of Chosen Pesticides on *A. mellifera* Health

Examples of possible effects of pesticides that are applied in agriculture and pose a threat to bee health have been collected in the table below (Table 1).

## 6. Bee Legal Protection against Pesticides

In beekeeping, the area in which colonies feed is important. In developed countries, a large part of the land is used for agricultural purposes, which is associated with the frequent use of large amounts of pesticides on the agenda. The use of these agents is necessary to protect plants from pests [148]. It shall be stressed that the efficient protection of bees requires adoption of the legal measures. International organizations as OECD adopted projects to harmonize the methods used by OECD countries to evaluate pesticides risks to health and the environment, however the OECD guidelines are only non-binding roadmaps for the OECD governments, without real legal binging force. Thus, the authors wish to refer to the legal system of the European Union as an example of the legally binding system established in reference to the control of pesticides. The European Union, as an international organization of 27 member states and over 445 million Inhabitants, is a unique example of the politic and economic integration which is in power to establish its own legal system. In the EU, the rule of supremacy of the EU law was established, which means that the legal acts of the EU prevail over the national legislation. 

Moreover, the legal system of the European Union, also in the reference of pesticides, has further direct impact on the worldwide regulations of the other international organizations and countries all over the world. Many legal rules, which were adopted on the level of the European Union, influenced the global discussion on climate change, protection of biodiversity, and the protection of health of human, animals, and plants. In the recent legal framework development of the EU, an especially important role is played by the European Green Deal, which is a long-term strategy which aims not only for climate-neutrality by the year 2050, with the significant reduction in greenhouse gas emissions, but also to reduce the use of pesticides by 50% by 2030 (Part “From Farm to Fork”), and to aid the pollination process by reversing the decline in pollinators (Part “Biodiversity”) [149].

Pesticide producers are legally obliged to determine if their products are harmful to bees. Regulation (EC) No 1107/2009 of the European Parliament and of the Council of 21 October 2009, concerning the placing of plant protection products on the market and repealing Council Directives 79/117/EEC and 91/414/EEC, and Directive 2009/128/EC of the European Parliament and of the Council of 21 October 2009, establishing a framework for Community action to achieve the sustainable use of pesticides, are the basic legal acts in the European Union regulating issues related to the integral protection of pollinating insects—including honey bees—against the negative effects of pesticides on their welfare [150,151].

It is worth mentioning that rules on pesticides were regulated by the legal act in the form of regulation, which is an expression of the highest level of European unification of the legal system, as the regulations are binding directly in all member states of the European Union, without the obligation of implementation into the national legal system. The directives are another type of the European secondary law, which sets out goals of legal regulation but allows member states to concretize measures for its implementation into national legal systems. Thus, the directives are measures of harmonization of legal systems within the European Union which are binding with the respect to the aim to be achieved. They are leaving some scope of flexibility to the European countries by way of the form and method of national implementation of its provisions. In practice, it means that member states’ varied legal acts could be adopted to achieve the goal stated in the directive, however, they could be significantly different from the national legal systems. 

In accordance with art. 2 clause 1 in connection with art. 3 point 10 lit. a) Directive 2009/128/EC, “pesticide” means a plant protection product as defined in Regulation (EC) No 1107/2009. This Regulation (EC) No 1107/2009 shall apply to products, in the form in which they are supplied to the user, consisting of, or containing active substances, safeners, or synergists, and intended for one of the following uses:

1. Protecting plants or plant products against all harmful organisms or preventing the action of such organisms, unless the main purpose of these products is considered to be for reasons of hygiene rather than for the protection of plants or plant products;
2. Influencing the life processes of plants, such as substances influencing their growth, other than as a nutrient;
3. Preserving plant products, in so far as such substances or products are not subject to special community provisions on preservatives;
4. Destroying undesired plants or parts of plants, except algae, unless the products are applied on soil or water to protect plants;
5. Checking or preventing undesired growth of plants, except algae, unless the products are applied on soil or water to protect plants.

These products are referred to as “plant protection products”.

Directive 2009/128/EC establishes a framework to achieve a sustainable use of pesticides by reducing the risks and impacts of pesticide use on human health and the environment, and promoting the use of integrated pest management and of alternative approaches or techniques such as non-chemical alternatives to pesticides, whereas Regulation (EC) No 1107/2009 lays down rules for the authorization of plant protection products in commercial form and for their placing on the market, use and control within the community, and also both rules for the approval of active substances, safeners, and synergists, which plant protection products contain or consist of, and rules for adjuvants and co-formulants. The purpose of this Regulation is to ensure a high level of protection of human and animal health and the environment, and to improve the functioning of the internal market through the harmonization of the rules on placing on the market plant protection products, while improving agricultural production. Those rules are underpinned by the precautionary principle in order to ensure that active substances or products placed on the market do not adversely affect human or animal health or the environment. In particular, member states shall not be prevented from applying the precautionary principle where there is scientific uncertainty as to the risks with regard to human or animal health or the environment posed by the plant protection products to be authorized in their territory.

The provisions of Regulation No 1107/2009, as compared to the previous ones, introduced a number of regulations that will ensure greater safety of using plant protection products for bees, including:

1. One of the criteria for the approval of active substances (as well as safeners and synergists) is to demonstrate that this substance does not cause significant exposure to honey bees, or cause unacceptable acute or chronic effects on the survival and development of honey bee colonies, including effects on bee larvae honey and honey bees behavior;
2. As a mandatory element of the documentation of the active substance, the applicant is required to submit reviewed, publicly available scientific publications on the active substance and its relevant metabolites, devoted to side effects on health, the environment and non-target species of this substance (including honey bees), in accordance with an indication of EFSA, published in the last 10 years before the date of submission of the dossier.

In addition, for the protection of bees, it is also important, from 2018, to prohibit the use of plant protection products in certain areas recognized as EFA, i.e., fallow land (including fallow land with honey-bearing plants), catch crops, nitrogen-fixing crops, and land strips for payment along the edges of the forest on which production takes place.

One of the reasons for CCD was the use of pesticides from the group of neonicotinoids, and in particular three active substances, i.e., imidacloprid, clothianidin, and thiamethoxam. In 2013, the Commission imposed restrictions on the application of the above mentioned three neonicotinoid pesticides [152] after they were shown to pose a significant risk to bees. In February 2018, EFSA confirmed the existence of these threats in a review of available evidence [153,154,155]. On 27 April 2018, member states supported the Commission proposal to further reduce the use of three neonicotinoid pesticides. As a result, as part of the implementation of the provisions of Commission Regulations No. 2018/783 and 2018/784 and, as of 19 September 2018, all authorizations for plant protection products containing active substances from the neonicotinoid group (imidacloprid, thiamethoxam, and clothianidin) were withdrawn [156,157,158]. Additionally, it is worth noting that when the EU banned the use of the three above-mentioned pesticides, France, out of concern for the protection of bees, has expanded this catalog by another two, i.e., thiacloprid and acetamiprid.

After the introduction of neonicotinoids in the late 1990s, they were promoted by statements about their effectiveness and limited side effects on organisms, including bees. However, afterwards, scientific research established a link between neonicotinoids and CCD [159,160,161]. The process of registering a new insecticide requires the production of detailed environmental risk assessments, which is regulated by two European legal acts: Commission Regulation (EU) No 283/2013 of 1 March 2013 setting out the data requirements for active substances, in accordance with Regulation (EC) No 1107/2009 of the European Parliament and of the Council concerning the placing of plant protection products on the market (OJ EU L 93/1) and Commission Regulation (EU) No 284/2013 of 1 March 2013, setting out the data requirements for plant protection products, in accordance with Regulation (EC) No 1107/2009 of the European Parliament and of the Council concerning the placing of plant protection products on the market (OJ EU L 93/85). At the time neonicotinoids were authorized, risk assessment schemes were inadequate to detect some of threats, as they were designed for “spray application” only. They were created without the consideration of evaluation of seed treated and soil-drenching chemicals and also assumed exposure to be restricted to the pesticide application period and to the treated crop [162]. Moreover, neonicotinoids were applied at low concentrations compared to other insecticides due to their high toxicity, and thus they were found in pollen and nectar at very low levels, so for many years the ability to detect them was limited by analytical sensitivity. In the procedure of authorization, pesticides undergo a risk assessment process aiming to assure the absence of unacceptable risks to the environment. The European Plant Protection Organization (EPPO) worked on the harmonization of test protocols and adopted a bee risk assessment scheme in 1999, which was revised in 2010. The risk assessment is structured in the form of the tiered approach. The first tier consists of a battery of cost-effective laboratory tests based on acute exposure and LD5 estimates. Products showing significant levels of toxicity are elevated to more environmentally relevant semi-field and field tests (tiers 2 and 3). It shall be stressed that sublethal effects can be detected in tiers 2 and 3 (field and semi-field), but absence of the high rate of mortality in tier 1 (laboratory) eliminates the neonicotinoids from submission to higher tiers.

At present, significant changes were introduced to the protocols of risk assessment, but in the scientific research it is stated that the procedures are still insufficient to assess some of the threats posed by pesticides. Thus, it is claimed that more holistic risk assessment should be adopted which considers temporal and spatial dimensions of pesticide exposure; co-exposure to multiple compounds; differences among bee species with different life histories in levels to exposure and intensity; and sublethal effects [163]. 

To protect bees properly, it might be beneficial to take the hours of their highest activity into consideration. Consequently, pesticides should be sprayed late in the evening, when the bee’s activity is the lowest [164]. Another way to avoid bees’ death from pesticides is to choose their right formulas. Pesticides come in the form of concentrates and solutions; they dry quickly and do not leave residues such as powders. In addition, dusts and powders stick to the hairs on the bees’ bodies, as a result of which they are transferred to the hive and stored with pollen. This creates a risk of colony collapse if contaminated pollen is given to the brood and to the queen. Beekeepers should also use pesticides that quickly degrade and are the least toxic. To protect bees from pesticides, apiaries should be set up a few miles from farmland due to the toxic substances sprayed there. The location of apiaries is one of the most important factors in the prevention of pesticide poisoning [165].

The authors are aware that the framework of the article does not allow for an exhaustive presentation of the theoretical and legal controversies related to the protection of the honey bee environment in the light of the creation and application of legal regulations within the European Union. However, even the “fragmentary” findings made in this publication resulted in an answer to the question of the legal situation in the field of broadly understood protection of bees in EU regulations. Undoubtedly, in-depth research on the presented matter of legal regulations is needed, as only a comparative approach, supported by reference to developed and effective legal mechanisms in various countries, will allow to fully capture the advantages and weaknesses and define the directions of their desired changes. However, these issues will be the subject of a separate study.

## 7. Microorganisms as One of the Factors Triggering CCD

Microorganisms are another factor that influence CCD and negatively affect the health of the entire colony. Bee-related microorganisms include bacteria, protists, and fungi, some of which are important bee pathogens. Microorganisms usually spread quickly due to beekeeping activities and can be fatal to bees when untreated. Treatment of some microorganisms is expensive and sometimes requires the sacrifice of hives and entire colonies.

### 7.1. Bacteria

#### 7.1.1. *Paenibacillus larvae*

*Paenibacillus larvae* are Gram positive, flagellated, spore-forming, and round-ended bacteria. They are specialized pathogens affecting bee larvae. Their size varies, reaching a width of 0.5 µm and a length of 1.5 to 6 µm [166]. *P. larvae* belong to relatively anaerobic bacteria (laboratory strains grow well under aerobic conditions). Bacteria isolated directly from the material grow best at 20–40 °C (optimum 35–37 °C) and in an atmosphere of 5–10% CO_2_ [167]. *P. larvae* do not produce catalase, do not hydrolyze casein or starch, and do not dissolve gelatin, nor reduce nitrates to nitrites [168]. 

*P. larvae* are considered to be the etiological agent of American foulbrood of honey bees, a disease affecting beekeeping in many world regions [169]. *P. larvae* are a lethal pathogen known to initiate and/or accelerate the onset of CCD. The progress of American foulbrood, even despite the removal of infected larvae by nurse bees, can lead to a colony collapse [170]. Spores of this bacterium can survive up to 50 years and are resistant to high temperatures, drought, and ultraviolet light [171]. They develop in the midgut of the larva. Tube cells are not able to proliferate and, with the help of flagella, penetrate the body cavity and multiply in hemolymph. Bee larvae fall victim to a systemic bacterial infection and die [172]. They break down into a brown, sticky liquid. The resulting scale dries out and contains a huge number of bacterial spores. Spores of *P. larvae* can also be isolated from honey, pollen, nectar, and the walls of the hive [173].

The spread of spores between colonies occurs through beekeeping and adult bees. Infection with about ten spores can already be fatal to bee larvae [174]. The high concentration of colonies next to each other favors the spread of the disease. Treatment of American foulbrood disease is very limited. Some antibiotics only affect the vegetative form of bacteria and antibiotic residues can be detected in bee products such as honey or propolis [175]. Regular monitoring of colony health is a significant factor in American foulbrood prevention and essential oils are often used to control it [172].

#### 7.1.2. *Melissococcus plutonius*

*Melissococcus plutonius* are anaerobic, Gram positive bacteria. They are lanceolate cocci and reach a size of about 0.5 × 1.0 µm. *M. plutonius* occurs singly, in clusters, and in longitudinal chains. In laboratory conditions, they are most favorable for an atmosphere of 5–10% CO_2_ at 35 °C. In vitro, *M. plutonius* are somewhat pleomorphic and often occur in rod form. These bacteria can be detected using a microscope, immunological methods (e.g., ELISA), or polymerase chain reaction [166].

*M. plutonius* is a biotic factor contributing to the abnormal CCD phenomenon that threatens bee health on a global scale [176]. This pathogen is an etiological agent of European foulbrood disorder [177]. European foulbrood is a disease affecting unsealed broods between 3 and 4 days of age [178]. Infection occurs by the larvae ingesting food containing bacteria. *M. plutonius* colonizes the larvae midgut. After infection, bee broods usually die after 4–5 days. The dying larva sticks to comb cells and turns brown until it eventually breaks down after death [177]. The European foulbrood disease is also influenced by other microorganisms that cause secondary infections in an already infected larva, affecting the symptoms of the disease. These microorganisms include *Paenibacillus alvei*, *Bacillus laterosporus*, *Enterococcus faecalis, M. plutonius* and *Enterococcus faecium* [179]. 

Some larvae can survive and excrete bacteria along with their feces. The disease spreads between colonies through drifting and robbing where bees are carriers of bacteria. Some individuals may survive to the pupal stage, however their weight will be reduced [180]. *M. plutonius* can also be detected in bee products such as honey. European foulbrood treatment is limited. The use of chemotherapeutics and antibiotics to treat bees from diseases has been banned. Residues of antibiotics could remain in bee products and after human consumption, negatively affecting health [181]. 

#### 7.1.3. *Serratia marcescens*

Opportunistic pathogens are a factor causing endogenous infections characteristic of individuals with reduced immunity and also as a result of taking antibiotics or immunosuppression. Many opportunistic pathogens are resistant to antibiotics, and it is often difficult to determine the factors leading to their pathogenicity due to their ability to live in environments other than host organisms [182]. Common opportunistic pathogens of bees are species from Enterobacteriaceae and Enterobacter, including *Serratia* [183]. 

*Serratia marcescens* is a nosocomial animal pathogen. It is a Gram negative, motile, rod-shaped, anaerobic bacterium. It usually occurs in water, on the surface of animals, in the digestive tract of animals and in the soil [184]. The red pigment (prodigiosin) produced by the *S. marcescens* probably plays a role in the breathing process. Bacteria from this species have low nutritional requirements and varying incubation temperatures ranging from 10 to 36 °C [185]. 

*S. marcescens* is only able to cause infection when it is present in the hemocoel, and can be detected in the guts of adult, weakened bees. Oral exposure to *S. marcescens* can lead to lethal infections. Bees weakened by other factors are more susceptible to infection with this opportunistic pathogen, which can lead to an acceleration of CCD [186]. It often causes death and sepsis [183]. *S. marcescens* does not induce the expression of antimicrobial peptides or phenoxide, suggesting that this bacterium has mechanisms to avoid the bees’ immune system [186].

### 7.2. Fungi

#### 7.2.1. *Ascosphaera apis*

*Ascosphaera apis* is heterothallic, spore-forming, filamentous fungus that infects bees [187]. Mature spores have an average size of 2 × 1.2 µm and are tightly packed in spherical spore balls that are about 8–16 µm in diameter. Over 10 spore balls form a spherical sporic cyst. Adult spores have two nuclei in different sizes. The larger nucleus is in the center of the spore, where the five times smaller nucleus is located at the end of the spore. Along the inner wall, various mitochondria are located, and the cytoplasm is filled with numerous ribosomes [188]. *A. apis* causes chalkbrood disease that attacks bee larvae [189]. Fungal spores germinate in an anaerobic hindgut environment. After mycelia develops, *Ascosphaera* sp. reaches the abdomen where it develops aerobically and then penetrates the cuticle. At first, the larva is spongy and white, but as the disease progresses, it hardens and becomes chalk-like. The sporocarps produced by this fungus develop on the body surface or integument of larvae and change their color to gray, black, or brown [190].

Chalkbrood disorder occurs in most regions of the world, and its clinical symptoms occur for a short period of time, usually during humid and cold weather conditions. Adult bee individuals are not exposed to this fungus, but they often transfer it between hives, causing even more colonies to become infected. Larvae become infected with the fungus by eating contaminated food [187]. *A. apis* can cause significant decrease in the number of bees and reduce their productivity. Chalkbrood is a problematic, chronic disorder and can persist in hives for a long time. *A. apis* feeds on the nutrients provided by the bee brood and, in some cases, can lead to colony collapse, especially if the bees are exposed to other stress-factors [191]. Chalkbrood disorder can be controlled by removing the diseased colony and using fungicides or natural products such as essential oils. To date, however, no compound has been found that would reach the appropriate level needed to combat this disease [190].

#### 7.2.2. *Aspergillus* sp.

*Aspergillus* sp. are filamentous, saprophytic fungi usually found in soil. The morphological identification of *Aspergillus* sp. is based on the study of criteria such as sexual or asexual structures and their characteristics, e.g., size, shape, color, attachment, and/or ornamentation [192]. They are also hydrophobic and thermotolerant, able to survive and develop at temperatures of 12–50 °C [193]. *Aspergillus* species are also able to produce the protease enzyme, which attacks protein peptide bonds [194].

Many *Aspergillus* species are stonebrood disorder agents. It is most often caused by *Aspergillus flavus*, less often by *Aspergillus fumigatus*. *Aspergillus niger* is a common agent that causes stonebrood disorder, but its role in causing the symptoms of the disease has not been determined [195]. Infection with *Aspergillus* species can provoke symptoms very similar to CCD and contribute to the collapse of entire bee colony [196]. The bee becomes infected with *Aspergillus* sp. through the gut after eating infected food. Infection can also be through the epidermis. Most *Aspergillus* sp. produces aflatoxins, which are probably the main cause of larvae death after infection [187]. *Aspergillus* sp. also occur saprophytically on beehive substrates, increasing the chance of infection by larvae. Hard, mummified larvae are a symptom of a disease that is visible in brood cells over time. *Aspergillus* sp. usually attacks individuals with weakened immunity and can cause a lot of damage to the colony [195].

#### 7.2.3. *Nosema* sp.

Parasitic fungi of the genus *Nosema* causes the dangerous bee disease nosemosis. In most cases it is caused by two species of microsporangia: *Nosema apis* and *Nosema ceranae* [197]. Both species occur in the external environment in the form of spores, and the vegetative form occurs only in the bee’s organism [198,199]. *Nosema ceranae* spores have a length of 3.3–5.5 µm and width of 2.3–3.0 µm. *Nosema apis* spores are longer by 1 µm and have a more regular shape. At 30 °C, the parasite’s development cycle lasts about 5 days [200].

*Nosema* sp. is microsporidian fungi, commonly found in CCD cases and is recognized as its potential causative agent [63]. Bees become infected by consuming water and food containing spores. Spores develop in the epithelial cells of the midgut, then the cell walls burst, and the spores are excreted with feces. Infected bees defecate in and around the hive, infecting other individuals [201]. PCR tests also detected the presence of *Nosema* species in salivary glands, hypopharyngeal glands, and fat body, but not in the muscles and brains of bees [202]. *Nosema ceranae* affects gene expression by changing hormonal and metabolic pathways. Oxidative stress in the bee’s body is increased, and the nutrient-sensitive structures become smaller [201]. Infected bees feel hungry and do not share food with the rest of the colony [203]. *Nosema* sp. negatively affect digestive tissue, weaken a bee and, in combination with other factors, lead to its death [204]. In addition, bioassays showed that some pesticides can enhance the mortality effect of *Nosema* sp. and increase food consumption of the bees, possibly caused by the fact that bees are trying to compensate for the energy lost by additional stress [205]. 

### 7.3. Varroa destructor

Species of *Varroa destructor* is an ectoparasitic mite attacking honey bee colonies. At first, it was mainly present in the Far East, but with the development of beekeeping, transport, and cosmopolitanization, it became a global problem [206]. Adult females reach a length of 1.0–1.77 mm and a width of 1.5–1.99 mm [207]. Male mites are smaller at all developmental stages [207].

Ectoparasitic mite *V. destructor* contributes to colony mortality and is often associated with CCD cases. Additionally, along with other factors that weaken bee health, it can restrict the growth of a bee colony and lead to its collapse [208]. It has such a huge impact on bee colonies by several factors. These mites are vectors of debilitating viruses, directly affect developing bees, and are ubiquitous in beehives. *V. destructor* lives outside its hosts. Female individuals occur attached to the bodies of adult bees and in worker cells, where reproduction occurs. *V. destructor* feed on the fat body tissue of adult and larval bees [209]. In addition, by weakening the body of bees (through blood loss), mites transfer pathogens to the bodies of their hosts [210]. The chemical control of *V. destructor* is often the most effective and economical. However, the mites become resistant to coumaphos, fluvalinate, and amitraz. This often results in overdosing of this pesticide and too high concentrations occurring in the colonies. In addition, bioassays showed that some pesticides can enhance the mortality effect of *Nosema* sp. and increase food consumption of the bees, possibly caused by the fact that bees are trying to compensate for the energy lost by additional stress [211].

## 8. Conclusions

Bees are an economically important species of pollinators due to their impact on agriculture around the world. Bee products such as honey, propolis, bee pollen, and royal jelly have a positive effect on human health due to their numerous properties. Bee-derived products are important due to their antioxidant, antimicrobial, anti-inflammatory, antiproliferative, and anticancer activity. They are also used in other sectors such as the beauty industry. Bee colony health is threatened by CCD, a phenomenon characterized by mass extinction of bees in the world. Due to the importance of bees to humans and the environment, this disease is at least worrying. It is believed that the causes of this phenomenon may be steam including pesticides, microorganisms, air pollution, and antibiotics. Pesticides are used in agriculture to combat pests harmful to crops and animals. However, some of them can be very harmful to the health of bees by affecting their nervous system and weakening bee resistance, contributing to the extinction of bees. Legal protection of bees, even adopted on the level of the European Law legislation, is not sufficient to assure the complex legal protection. Risk assessment procedures in authorization of pesticides should assure absence of unacceptable risks to the environment and take under consideration the direct and indirect negative impact on pollinators. Microorganisms, including fungi, bacteria, and mites, are another trigger for CCD. They cause various diseases in bee colonies, infecting larvae, adults, and the queen, often leading to the extinction of the entire colony. Microorganisms are spreading quickly, and beekeepers often have to go as far as eliminating the beehive after detecting microbial infection by bees to protect the entire apiary. Due to the hazardous effects of pesticides, in order to protect crops, the focus should be on the use of agents that do not endanger bee colonies. One should also consider the more frequent use of harmless bee preparations that would protect the colony against the influence of microorganisms dangerous to them, and this is a challenge for future research.

## Figures and Tables

**Table 1:** Recognized possible effects of chosen pesticides on *A. mellifera* health (under laboratory conditions).

Pesticide	Application	Possible Effect on Bees	References
Abamectin	Control of insect pests and mites destroying horticultural and agricultural crops.	Negatively affects the viability and cytotoxic midgut cells which can lead to digestive diseases.	[119,120]
Acephate	Control a wide range of chewing and sucking insect pests threating agricultural crops.	Suppresses esterase activity and reduces body weight.	[121,122]
Amitraz	Control of ticks, mites, and lice on domestic animals.	Increases mortality, leads to behavioral changes in adult honey bees.	[123,124]
Bifenazate	Control of spider mites.	Affects the physiology and behavior.	[125,126]
Bifenthrin	Control of insect pests, treatment of mosquito nets, suppression of malaria transmission.	Increases mortality, affects central and peripheral nervous systems.	[127,128]
Chlorfenapyr	Control of insect pests threating agricultural crops and animals.	Increases mortality, causes paralysis, affects nervous system.	[129,130,131]
Coumaphos	Control of insect pests threating agricultural crops and animals.	Reduces foraging activity, affects colony mortality, and affects the size of hypopharyngeal glands, significantly affects oxidative status.	[105,132,133]
Cypermethrin	Control of insect pests threating agricultural crops.	Leads to a significant hypoglucosemia and hypotrehalosemia, causes minor expressional changes of genes.	[134,135]
Deltamethrin	Control of disease vectors and eradicating unwanted insects.	Interferes with the nervous system such as memory-related characteristics and dance behavior.	[136,137]
Diazinon	Control of household insects and insect pests threating agricultural crops.	Reduces activity of acetylcholineasterase (an enzyme essential to the transmission of nerve impulses), affects odor learning.	[138,139]
Dimethoate	Control of insect pests threating agricultural crops.	Leads to inhibition of the acetylcholinesterase, affects physiological traits.	[140]
Fenitrothion	Control of insect pests threating agricultural crops.	Leads to a significant hypoglucosemia and hypotrehalosemia, inhibits acetylcholinesterase activity.	[134]
Fipronil	Control of insect pests threating agricultural crops (mostly sunflowers).	Increases mortality, affects foraging intensity and homing success.	[141]
Neonicotinoids	Control of insect pests threating agricultural crops and deterring pests on domesticated animals.	Increases mortality, affects central nervous system, increases the levels of deformed wing virus, affects a large number of up and down-regulated differentially expressed genes, and reduces standard metabolic rate in queens.	[142,143,144,145]
Spinosad	Control of insect pests affecting agricultural crops.	Absence of significant impact on honey bee colonies	[146,147]

## Data Availability

Not applicable.
